# Can a Single Session of a Community-Based Group Exercise Program Combining Step Aerobics and Bodyweight Resistance Exercise Acutely Reduce Blood Pressure?

**DOI:** 10.2478/hukin-2014-0089

**Published:** 2014-11-12

**Authors:** Romeu Mendes, Nelson Sousa, Nuno Garrido, Braulio Cavaco, Luís Quaresma, Victor Machado Reis

**Affiliations:** 1 Research Center in Sports Sciences, Health Sciences and Human Development; Portugal.; 2 University of Trás-os-Montes e Alto Douro; Vila Real, Portugal.

**Keywords:** physical activity, post-exercise hypotension, cardiovascular diseases, health promotion

## Abstract

This study aimed to analyze the acute effects of a single session of a community-based group exercise program combining step aerobics and bodyweight resistance exercise on blood pressure in healthy young adult women. Twenty-three healthy young adult women (aged 31.57 ± 7.87 years) participated in two experimental sessions (exercise and control) in a crossover study design. Blood pressure was monitored before, immediately after and at 10, 20 and 30 min of recovery. The exercise session consisted of four phases: 1) a warm-up (5 min of dance aerobics); 2) aerobic exercise training (30 min of step aerobics); 3) resistance exercise training (six sets of 12 repetitions of three bodyweight exercises in a circuit mode, 10 min); and 4) a cool-down (5 min of breathing and flexibility exercises); totaling 50 min of duration. Systolic blood pressure after exercise was significantly lower compared to control at the 10th min (−10.83 ± 2.13 vs. −2.6 ± 2.13 mmHg; p = 0.009), 20th min (−11.26 ± 2.13 vs. −3.04 ± 2.13 mmHg; p = 0.009) and 30th min of recovery (−10.87 ± 2.39 vs. −0.48 ± 2.39 mmHg; p = 0.004). A single session of a community-based group exercise program combining step aerobics and bodyweight resistance exercise was effective in inducing significant post-exercise hypotension in healthy young adult women. This type of low-cost exercise interventions may have an important role in the prevention of cardiovascular diseases and in community health promotion.

## Introduction

Cardiovascular diseases are a major cause of mortality and morbidity worldwide and high blood pressure (BP) is one of the main risk factors (World Health Organization, 2007; Mendes and Themudo Barata, 2008; Perk et al., 2012). Regular physical activity and exercise are widely recommended for the prevention and treatment of hypertension as a non-pharmacological strategy (Mancia et al., 2007). International recommendations advise a minimum of 30 min of continuous or accumulated moderate intensity aerobic exercise, on most, preferably all, days of the week, supplemented by resistance exercises (Pescatello et al., 2004; Mendes et al., 2011).

Post-exercise hypotension is a phenomenon of reduction in resting BP in the time following a single bout of exercise and has great clinical relevance as it can act as a non-pharmacological agent in preventing and treating high BP (MacDonald, 2002). Although aerobic exercise is one of the most usual recommendations for acute and chronic control of BP (Pescatello et al., 2004; Mancia et al., 2007; Mendes et al., 2013b), recent studies have also demonstrated the resistance exercise effectiveness in decreasing BP after a single bout of exercise (Morais et al., 2011; Tibana et al., 2013) and after a regular resistance exercise program (Cornelissen et al., 2011; Moraes et al., 2012).

Due to the potential cardiovascular, metabolic and musculoskeletal benefits of both types of exercise, combined aerobic and resistance exercise is becoming an emergent exercise mode that is used as a therapeutic tool in major non-communicable chronic diseases and in aged populations (Cao et al., 2007; Ho et al., 2012). This type of exercise training seems to be the most used in community-based exercise programs developed by institutions all over the world aiming to improve health outcomes and physical fitness (Mendes et al., 2013a; Plotnikoff et al., 2013). Community-based exercise programs are recommended by the World Health Organization (2009) as effective strategies of health promotion and disease prevention. However, few studies have analyzed the effect of a single bout of combined aerobic and resistance exercise in BP values, which makes this a scarce and poorly studied area, considering the diverse possible exercise protocols and different individual characteristics and responses (Keese et al., 2011; Ruiz et al., 2011). Therefore, this study aimed to analyze the acute effects of a single session of a community-based group exercise program combining step aerobics and bodyweight resistance exercise program on BP in healthy young adult women.

## Material and Methods

### Study Design

This was a crossover study. Study sample underwent two experimental sessions (exercise and control), one week apart, with subsequent BP monitoring for 30 min. The conditions for the study protocol were the same for the exercise and control sessions.

### Participants

Twenty-three healthy young adult women (aged 31.57 ± 1.64 years; body mass index 23.35 ± 0.94 kg/m^2^; waist circumference 80.26 ± 1.70 cm; clinical systolic BP 109.23 ± 2.50 mmHg; clinical diastolic BP 71.21 ± 2.05 mmHg) participating in a community-based group exercise program (at least for two months), not taking medications that influenced BP or the heart rate, and non-smokers, volunteered for this study. This study was conducted in accordance with the principles defined in the *Declaration of Helsinki* and was approved by the *University of Trás-os-Montes e Alto Douro Research Ethics Committee* (Vila Real, Portugal). All participants signed an informed consent term form prior to the beginning of the study.

### Experimental Protocol

Individual’s clinical BP was measured within three successive weeks according to the standards of the European Society of Hypertension (O'Brien et al., 2003) with a clinically validated automatic BP monitor (*Omron M6 Comfort*, Japan). One week before experimental sessions, participants were familiarized with the exercise protocol and recovery BP measuring procedures. Participants were instructed not to consume alcohol, coffee, or chocolate within five hours before experimental sessions and not to perform exercise within 24 hours before the measurements.

Participant’s baseline BP was measured after a period of 10 min seated rest, before each experimental session. Exercise session consisted of a group fitness class from a community-based exercise program. This was a supervised low-cost exercise program, developed only with fitness step platforms and fitness mats. The exercise session comprised four phases: 1) a warm-up (5 min dance aerobics); 2) aerobic exercise (30 min step aerobics with two different choreographies performed separately and together); 3) resistance exercise (6 sets of 12 repetitions of 3 bodyweight exercises: push-ups, isometric front planks, and lunges, in a circuit mode, 10 min); and 4) a cool-down (5 min of breathing and flexibility exercises); totaling 50 min of exercise duration. Step platforms were 15 cm high and movement cadence was defined by music with 128–132 beats per minute. Participants were asked not to perform Valsalva maneuver during resistance and flexibility exercises. Exercise intensity was controlled using the Borg’s Rating of Perceived Exertion Scale (Borg, 1982), already in use in the community-based group exercise program. Individuals were asked to assess their effort at four moments during the exercise session: 1) at the end of the first step aerobics choreography; 2) at the end of the second step aerobics choreography; 3) at the end of the gathering of both step aerobics choreographies; and 4) at the end of the resistance exercises. The control session consisted of sitting at rest for 50 min. BP was measured immediately after both experimental sessions (zero min) and at the 10^th^, 20^th^ and 30^th^ min of seated recovery.

Participants were instructed to drink water as desired (*ad libitum*) at baseline, during experimental sessions and recovery period, in order to avoid the potential effect of dehydration on BP responses.

### Statistical Analysis

Variation of systolic blood pressure (SBP) and diastolic blood pressure (DBP) between baseline and recovery (0, 10^th^, 20^th^ and 30^th^ min) was calculated for each participant, in both sessions (exercise and control). The SBP and DBP responses were compared between sessions by a two-way ANOVA with repeated measures, establishing sessions (control and exercise) and time (0, 10^th^, 20^th^ and 30^th^ min of recovery) as the main factors. Post-hoc comparisons were analyzed by the Bonferroni Test. Statistical significance was set at *p* < 0.05. Results are presented as Mean ± SD. All statistical analyses were conducted with PASW^®^ Statistics 18 for Windows*^®^*.

## Results

**Figure 1:**
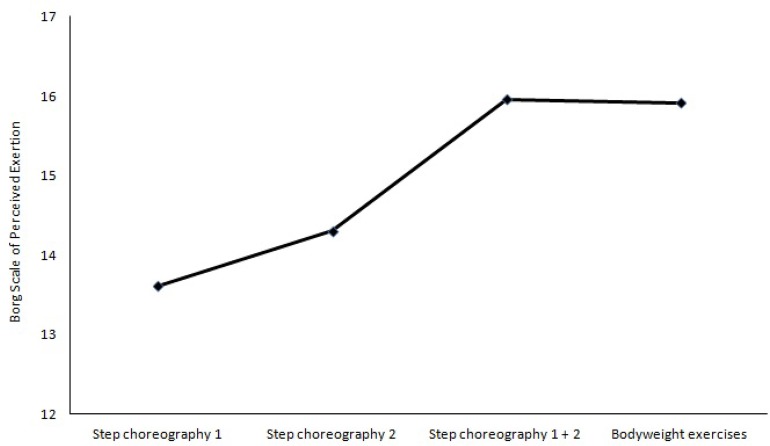
Graphic representation of exercise intensity in different phases of an exercise session according to the Borg’s Rating of Perceived Exertion Scale

Exercise intensity values during the exercise session are presented in Figure 1. Intensity was classified as vigorous [14 to 17 on the Borg’s scale (Garber et al., 2011)] at all moments of assessment.

**Figure 2:**
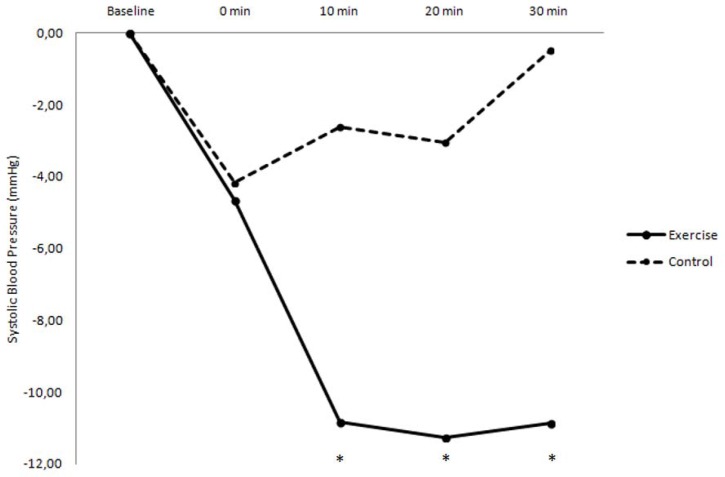
Variation of systolic blood pressure values between baseline and recovery (0, 10^th^, 20^th^ and 30^th^ min) in both experimental sessions (exercise and control). * significant difference (p < 0.05) between exercise and control

ANOVA indicated significant differences in SBP variations at the 10^th^ (−10.83 ± 2.13 vs. −2.61 ± 2.13 mmHg; *p* = 0.009), 20^th^ (−11.26 ± 2.13 vs. −3.04 ± 2.13 mmHg; *p* = 0.009) and 30^th^ min of recovery (−10.87 ± 2.39 vs. −0.48 ± 2.39 mmHg; *p* = 0.004), between exercise and control sessions (Figure 2). No differences were found in SBP variations immediately after the experimental sessions (−4.65 ± 3.27 vs. −4.17 ± 3.27 mmHg; *p* = 0.918).

**Figure 3:**
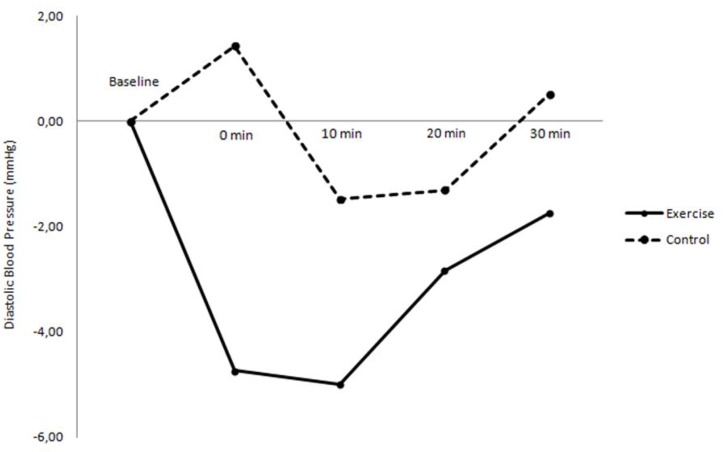
Variation of diastolic blood pressure values between baseline and recovery (0, 10^th^, 20^th^ and 30^th^ min) in both experimental sessions (exercise and control)

Although the values of DBP after exercise were always inferior to control, no significant differences were identified in DBP variations at 0 (−4.74 ± 2.33 vs. 1.43 ± 2.33 mmHg; *p* = 0.068), 10^th^ (−5.00 ± 1.53 vs. −1.48 ± 1.53 mmHg; *p* = 0.110), 20^th^ (−2.83 ± 1.88 vs. −1.30 ± 1.88 mmHg; *p* = 0.570) and 30^th^ min of recovery (−1.74 ± 2.23 vs. 0.52 ± 2.23 mmHg; *p* = 0.478), between exercise and control sessions (Figure 3).

## Discussion

The main finding of this study was that a single session of a community-based group exercise program combining step aerobics and bodyweight resistance exercise program was effective in inducing significant post-exercise hypotension in healthy young adult women, especially through SBP reduction.

This is an understudied area and to the best of our knowledge only two studies available in scientific literature have analyzed the acute effects of a single session of combined aerobic and resistance exercise on post-exercise BP (Keese et al., 2011; Ruiz et al., 2011). Studies of Keese et al. (2011) and Ruiz et al. (2011) reported post-exercise hypotension after a session of combined exercise, but the aerobic and resistance training protocols that were used were much different than those in the present study. Indeed, the authors used cycle ergometers for aerobic exercise and resistance machines for strength exercise, while in the current study low-cost material resources (fitness step platforms and mats) were used. This type of low-cost exercise programs has already shown to chronically reduce BP and improve health profile (Kraemer et al., 2001). Independently of the exercise protocol, combining aerobic and resistance training seems to be effective in inducing significant post-exercise hypotension.

Exercise intensity is one of the prescription variables that can affect post-exercise hypotension. Available evidence has suggested that vigorous exercise, when compared to activities of lower intensities, induces post-exercise hypotension with higher magnitude and duration (Quinn, 2000; Simao et al., 2005). One recognized vasodilatory phenomenon during exercise recovery is immediate post-exercise hyperaemia, which appears to be dependent on exercise intensity (Halliwill et al., 2013). In this study, exercise intensity was classified as vigorous at all moments assessed, with higher values at the final of step aerobics training and during bodyweight resistance training. The Borg’s scale was used to assess exercise intensity because all participants were familiarized with this method for at least three months. Other studies have also applied high intensity exercise but used heart rate monitors and one-repetition maximum strength testing for controlling aerobic and resistance exercise intensity, respectively (Keese et al., 2011; Ruiz et al., 2011). In fact, higher exercise intensities are recommended as a key element to obtain major cardiovascular benefits as long as there is no risk of cardiovascular or orthopedic trauma (Thompson et al., 2003; Haskell et al., 2007; Sousa et al., 2013).

The participants in the current study were apparently healthy young adult women, participating in an exercise program twice a week at least for three months, normotensive, non-smokers and without other known major cardiovascular risk factors. These inclusion criteria enabled a safe application of high intensity exercise. Although the phenomenon of post-exercise hypotension is well established in hypertensive and normotensive individuals, greater hypotensive effects are observed in individuals with higher baseline levels (Pescatello et al., 2004; Casonatto and Polito, 2008). Our data cannot support such an assumption since the participants had clinical BP levels classified as optimal according to the European Society of Hypertension and the European Society of Cardiology (Mancia et al., 2007). Hence, it is possible that other individual characteristics such as gender, age, body composition, physical fitness and regular physical activity could have influenced the obtained results. Nevertheless, the combination of aerobic and resistance exercise and high intensity maintained through all sessions are likely to be the factors with most influence on the acute BP reduction observed in the present study.

Post-exercise BP was measured only 30 min after the experimental sessions, what could be viewed as a limitation of the study. In fact, more prolonged BP monitoring could have confirmed higher duration and magnitude of post-exercise hypotension as earlier reported (Keese et al., 2011; Ruiz et al., 2011). Future studies should use ambulatory BP monitoring to assess post-exercise BP and analyze the influence of different sequences in combining aerobic and resistance exercise.

From the results of this study we can conclude that a single session of a community-based group exercise program combining step aerobics and bodyweight resistance exercise was effective in inducing significant post-exercise hypotension in healthy young adult women. This type of a low-cost exercise intervention seems to have sufficient intensity to trigger benefits in cardiovascular health and its regular practice may play an important role in the prevention of hypertension and in community health promotion.
